# Editorial Note: Does menstrual hygiene management and water, sanitation, and hygiene predict reproductive tract infections among reproductive women in urban areas in Ethiopia?

**DOI:** 10.1371/journal.pone.0335092

**Published:** 2025-10-22

**Authors:** The PLOS One Editors

This article [1] was identified as one of a series of articles for which the PLOS One Editors have concerns about authorship and adherence to the journal’s first and second publication criteria. We regret that the issues were not addressed prior to the article’s publication.

Contrary to the article’s Data Availability statement, the original raw data files supporting the article’s results were not provided with the article. The Data Availability statement is updated to: All relevant data are available from https://doi.org/10.7910/DVN/VYMRCW.

The authors provided additional information during editorial follow-up to clarify the contents of the dataset and calculation methods used in generating results. This information is provided with this notice as S3 File and S4 File respectively.

## Supporting information

S3 File Detailed description of each column header in the underlying data. (DOCX)

S4 File Instructions for generating the results presented in columns CB through CU of the underlying data. (DOCX)
