# Supplementary material for: Editorial Note: Does menstrual hygiene management and water, sanitation, and hygiene predict reproductive tract infections among reproductive women in urban areas in Ethiopia?
Source: PLoS One. 2025 Oct 22;20(10):pone.0335092.exml. doi: 10.1371/journal.pone.0335092 (PMC12543146; doi:10.1371/journal.pone.0335092)
Supplement: S3 File — (DOCX) [file pone.0335092.s001.docx]

| COLUMN | ABBREVIATION | DESCRIPTION |
| --- | --- | --- |
| B | AGE | Age of respondents |
| C | MARITAL | Marital status of respondents |
| D | RELIGION | Religion of respondents |
| E | EDUSTAT | Educational status of respondents |
| F | NOPARITY | Number of parity for respondents |
| G | LINEWATE | houseline water(no/yes) |
| H | BONOWATE | Bono water (no/yes) |
| I | TOILET | what type of toilet do you use |
| J | OWNERHOU | who is the owner of your living house |
| K | CLASS | does your living house have dividing class |
| L | BEDROOM | do the house have separate bedroom |
| M | KITCHEN | do you have separate kitchen |
| N | GROUND | the floor is natural ground |
| O | WOOD | the floor is wood |
| P | CEMENT | the floor is cement |
| Q | WALLWOOD | the wall is wood but not have mod |
| R | WALLWOMO | The wall is wood with mod |
| S | WALWOCEM | The wall is wood and cement |
| T | WALBLOCK | The wall is blocket |
| U | ELECTRIC | Does the house have electric city system |
| V | GAS | Does the house use gas/kerosene as energy source |
| W | WOODLEAF | Does the house use wood/leaf as energy source |
| X | CHARCOAL | Does the house use charcoal as energy source |
| Y | RADIO | Do you have radio |
| Z | TV | Do you have TV |
| AA | FRIDGE | Do you have fridge |
| AB | CHAIR | Do you have chair |
| AC | TABLE | Do you have table |
| AD | BED | Do you have bed |
| AE | CYCLE | Do you have cycle |
| AF | MOTRCYCL | Do you have motor cycle |
| AG | BAJAJI | Do you have Bajaji/car |
| AH | BANKBOOK | Do you have bankbook |
| AI | LATPRISH | is the latrine shared or private |
| AJ | UTILIZE | is the latrine utilized |
| AK | CLEAN | cleanliness of latrine/toilet |
| AL | DISTANCE | distance of latrine from the house |
| AM | AMOWATER | amount of water used in l/c/d |
| AN | HANDWASH | hand washing with soap before touching the genital area |
| AO | WATERTOI | presence of water near toilet/latrine for hand washing |
| AP | H2OSOURC | What is your source of water? |
| AQ | ABORTION | history of abortion |
| AR | PREVIOUS | symptom of RTI in the past one year before 3 month of the data collection |
| AS | MULTIPLE | multiple sexual partner |
| AT | SEXINTER | sexual intercourse during menstruation |
| AU | CONTRAE | type of contraceptive used |
| AV | ABSORBENT | did you used blood absorbent material during menstruation |
| AW | NATUREAM | nature of absorbent material used during menstruation |
| AX | PAD | where do you put your menstruation pad/cloth (if you reuse) |
| AY | PLACEOFD | place of drying for washed reusable absorbents |
| AZ | FREQDAY | frequency of the day for changing of absorbent material |
| BA | FREINDY | frequency of changing of absorbent material per day |
| BB | BODYBATH | did you take all body bath every day during menstruation |
| BC | BATHDAT | when you take all body bath during menstruation |
| BD | WASHGENI | did you wash the genital area every day during menstruation(if yes) |
| BE | FREWASHG | how many times did you wash genital area per day |
| BF | USEDTOWA | what do you use to wash genital area |
| BG | VDISCHAR | yellowish or greenish or white type with burning sensation |
| BH | BURNURIN | burning sensation during urination |
| BI | ITCHING | itching/irritation around the genitalia |
| BJ | BACKPAIN | lower back pain |
| BK | ABDOPAIN | lower abdominal pain |
| BL | ULCER | genital ulcer/lesion |
| BM | RTI | reproductive tract infection |
| BN | FLUSHT | Flush toilet |
| BO | TRADIT | Traditional toilet |
| BP | VIPL | ventilated improved pit latrine |
| BQ | OWNER | owner of house |
| BR | BATHDAY1 | day of taking all body bath during menstruation days |
| BS | FREWASG1 | frequency of washing the genital area per day |
| BT | PARITY | number of parity categorized |
| BU | AMOUNTH2O | Amount of water categorized |
| BV | PROXIMITYL | proximity of latrine categorized |
| BW | AGECAT | age category |
| BX | EDUCAT | educational status categorized |
| BY | RELIGIONCAT | religion of respondents categorized |
| BZ | IUCD | use of IUCD |
| CA | WATERSOURCE | source of water |
| CB | FAC1_1 | Factor score 1 generated from PCA analysis |
| CC | FAC2_1 |  |
| CD | FAC3_1 |  |
| CE | FAC4_1 |  |
| CF | FAC5_1 |  |
| CG | FAC6_1 |  |
| CH | FAC7_1 |  |
| CI | FAC8_1 |  |
| CJ | FAC9_1 |  |
| CK | FAC10_1 |  |
| CL | ZFAC1_1 | Standardized factor score 1 to generated from PCA analysis |
| CM | ZFAC2_1 |  |
| CN | ZFAC3_1 |  |
| CO | ZFAC4_1 |  |
| CP | ZFAC5_1 |  |
| CQ | ZFAC6_1 |  |
| CR | ZFAC7_1 |  |
| CS | ZFAC8_1 |  |
| CT | ZFAC9_1 |  |
| CU | ZFAC10_1 |  |
| CV | WEALTHSCORE |  |
| CW | RWEALTHS | Rank of wealth score |
| CX | NWEALTHS | Percentile Group of wealth score |
| CY | RAN001 | Rank of wealth score |
| CZ | NTI001 | Percentile Group of wealth score |
| DA | EWI3G | Economic wealth index 3 category |
| DB | EWI5G | Economic wealth index 5 category |
| DC | FREQDAYCAT | frequency of the day for changing absorbent material category |
| DD | FREINDYCAT | frequency of changing absorbent material per day during menstruation category |
