# Supplementary material for: Editorial Note: Does menstrual hygiene management and water, sanitation, and hygiene predict reproductive tract infections among reproductive women in urban areas in Ethiopia?
Source: PLoS One. 2025 Oct 22;20(10):pone.0335092.exml. doi: 10.1371/journal.pone.0335092 (PMC12543146; doi:10.1371/journal.pone.0335092)
Supplement: S4 File — (DOCX) [file pone.0335092.s002.docx]

We used variables from column G to AH and from column BN to BQ to analyze wealth index using the principles of Principal component analysis (PCA). We Used SPSS for data analysis.

NOTe: for PCA analysis the question should be labeled with no value of 0 and yes value of 1 thus column the VARIABLES used for PCA analysis is LINEWATE BONOWATE CLASS BEDROOM KITCHEN GROUND WOOD CEMENT WALLWOOD WALLWOMO WALWOCEM WALBLOCK ELECTRIC CHARCOAL RADIO FRIDGE CHAIR TABLE BED CYCLE MOTRCYCL BAJAJI BANKBOOK flushT tradiT owner

The steps for analysis is as follows:

1. Open the dataset using SPSS
2. From SPSS menu go to analyze _______dimension reduction________factor_____ then select the variables from the list and transfer to the variables box
3. Then click on descriptive then click initial solution and KMO and Bartlett's Test box then continue
4. Then click on extraction then method the default Principal components, from analyze box click on correlation matrix, from display box click on unrotated factor solution, from extract box click on based on eigenvalue greater than 1 then continue
5. Then click on Rotation then click on varimax then continue
6. then we click ok
7. then we check the assumption of PCA analysis

here we see: the value of Kaiser-Meyer-Olkin Measure of Sampling Adequacy (KMO) should be greater than 0.5 and Bartlett's Test significance should be less than 0.05

Communalities table variables with extraction value less than 0.5 should be removed from the analysis stepwise until the value of each variable have greater than 0.5.

1. Then click on scores, then click on save as variables, here the system saves 10 principal components based on Eigen value greater than 1.
2. Then we have to transform to each FAC values to standardized Z FAC values using the menu transform then clicking compute variables for each of the generated 10 principal components. This is because we use to fulfill the assumption of logistic regression.
3. Then we compute wealth score by clinking transform then compute variable using this numeric expression

“ ZFAC1_1 * FAC1_1+ ZFAC2_1 * FAC1_1+ ZFAC3_1 * FAC1_1+ ZFAC4_1 * FAC1_1+ ZFAC5_1 * FAC1_1 + ZFAC6_1 * FAC1_1+ ZFAC7_1 * FAC1_1 + ZFAC8_1 * FAC1_1+ ZFAC9_1 * FAC1_1+ ZFAC10_1 * FAC1_1 ”

This is because we have ten principal components. Then after we classify to 5 or 3 category of wealth score. These were done clicking transform then clicking Rank cases by entering wealth score into the variable box.
